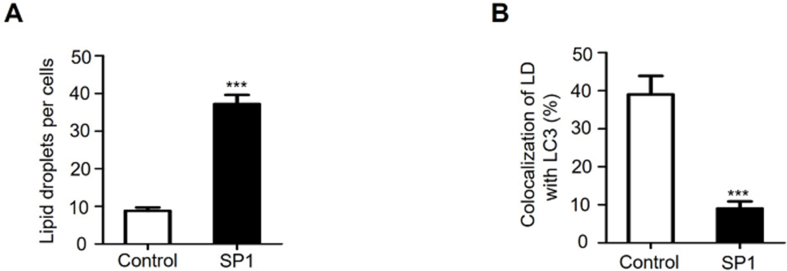# Regulation of *de novo* lipogenesis and lipophagy by *SP1* gene variants

**DOI:** 10.1016/j.gendis.2025.101722

**Published:** 2025-06-27

**Authors:** Soo Yeon Kim, Hyo-Jeong Ban, Siwoo Lee, Hee-Jeong Jin

**Affiliations:** Korean Medicine (KM) Data Division, Korea Institute of Oriental Medicine, Daejeon 34054, Republic of Korea

Lipid metabolism abnormalities are influenced by external and internal factors. Cold hypersensitivity refers to experiencing a cold sensation when the environment is not considered cold, and is clinically closely related to dyslipidemia (see supplementary background).^1^ Specificity protein 1 (SP1) is a key transcriptional activator and genetic factor associated with cold sensitivity (CS),^2^ and it contributes to lipogenic pathways^3^ and regulates the autophagy process^4^; nonetheless, its role in lipid metabolism in CS is currently unclear. Herein, we aimed to explore the roles of *SP1* gene variants associated with CS in lipid metabolism. Using Mendelian randomization, we confirmed significant genetic causality between *SP1* and lipid metabolism parameters, including increased total cholesterol, low-density lipoprotein-C, and triglyceride levels, and reduced high-density lipoprotein-C levels. Variant alleles at *SP1* functional polymorphisms resulted in up-regulated transcriptional activation of mechanistic target of rapamycin (*mTOR*) and sterol regulatory element-binding transcription factor 1 (*SREBP1*) and down-regulated lipophagy. We thus revealed that *de novo* lipogenesis and lipophagy are regulated by gene variants of *SP1*. These results provide insights into the diagnosis and prediction of treatment response in patients with abnormal lipid metabolism.

Using single-nucleotide polymorphisms associated with CS (Fig. S1) as instrumental variables in Mendelian randomization analysis, we investigated the potential causal relationship between CS and lipid traits (Fig. S2A). We examined the influence of the regulating tagSNP (rs11170510) of SP1 on lipid metabolism traits (see supplementary materials and methods). Our analysis revealed a significant causal relationship between lipid metabolism (nonalcoholic fatty liver disease, NAFLD) and variations in SP1. We then used Mendelian randomization to analyze the effect of CS on NAFLD as an instrumental variable; results revealed that CS positively affected NAFLD (Estimate = 0.051; standard error = 0.024; 95% confidence interval, 0.005 to 0.097; *P* = 0.031) (Fig. S2C, D). These findings suggest a causal relationship between genetic inhibition of CS and altered lipid levels. It was confirmed, via ENCODE tools, that mTOR and SREBP1 were among the lipid metabolism genes regulated by SP1 (Fig. S3A, S4A). A chromatin immunoprecipitation assay revealed that SP1 was significantly enriched in the promoter region of *mTOR* (Fig. 1A)*. mTOR* promoter activity was enhanced by SP1 binding in HepG2 cells (Fig. 1B). Real-time reverse-transcription PCR in HepG2 cells showed that *mTOR* mRNA levels were significantly increased by SP1 (Fig. 1C). mTOR expression was up-regulated in the peripheral blood mononuclear cells (PBMCs) of the Korean Medicine Daejeon Citizen Cohort (KDCC) cohort with variant alleles, which increased *SP1* mRNA levels compared with those in PBMCs with reference alleles (Fig. 1D). Furthermore, we investigated whether SREBP1 was concurrently regulated by SP1 in the mTOR-SREBP1 signaling pathway during *de novo* lipogenesis. SP1 occupied the promoter region of *SREBP1* and up-regulated the transcriptional activity of *SREBP1* in HepG2 cells (Fig. 1E, F). *SREBP1* mRNA levels substantially increased following SP1 overexpression in HepG2 cells (Fig. 1G). *SREBP1* mRNA levels were enhanced more in PBMCs harboring variant alleles of *SP1* than in those harboring the reference alleles (Fig. 1H). Acetyl-CoA carboxylase alpha (*ACACA*), stearoyl-CoA desaturase (*SCD*), and fatty acid synthase (*FASN*) mRNA levels were enriched in PBMCs harboring *SP1* variant alleles (Fig. S4B). Furthermore, the ENCODE ChIP-seq dataset confirmed significant SP1 occupancy in the promoter regions of ACACA, SCD, and FASN (Figs. S5A, S5B, S5C). These results suggest that the lipogenic pathway genes are regulated by *SP1* gene variants. Our results show that SP1 regulated mTOR expression. We then investigated whether SP1 affected autophagy. *SP1* knockdown considerably increased the number of autophagic vesicles (LC3A/B puncta) compared with that under control conditions in HepG2 cells (Fig. S6A, S6B). siSP1 caused a significant enhancement in the levels of the lysosomal marker lysosomal-associated membrane protein 2 (LAMP2) in HepG2 cells compared with those in control cells (Fig. S6C, S6D). SP1 was overexpressed in HepG2 cells, and mRNA expression of autophagy-related 5 (*ATG5*), beclin 1 (*BECN1*), autophagy-related 16-like 1 (*ATG16L1*), and microtubule-associated protein 1 light chain 3 beta (*MAP1LC3B*) was down-regulated (Fig. 1I). *ATG5*, *BECN1*, *ATG16L1*, and *MAP1LC3B* mRNA levels in PBMCs with the variant allele were down-regulated compared with those in PBMCs with the reference allele (Fig. 1J). These results suggest that *SP1* is associated with down-regulated autophagy activation and transcriptional activity of autophagy-related genes according to the variant alleles. We confirmed that SP1 overexpression regulated *mTOR* and *SREBP1* expression. We thus investigated whether SP1 overexpression induced lipid accumulation in cells. SP1 overexpression in HepG2 cells significantly increased the number of lipid droplets (Fig. 1K; Fig. S7A). siSP1 up-regulated the levels of light chain 3 (LC3) puncta and LAMP2 protein (Fig. S6A, S6C), and that the expression of autophagy genes was reduced by increased expression of SP1 associated with the *SP1* variant alleles (Fig. 1J). We confirmed that, in HepG2 cells harboring the *SP1*-overexpression plasmid, co-localization of lipid droplets and autophagosomes was significantly decreased compared with that in cells harboring the control plasmid, and droplets increased by SP1 overexpression demonstrated a low rate of removal by autophagy (Fig. 1L; Fig. S7B). Our clinical results also showed that TG levels increased in cases with the increased expression-associated SP1 variant allele (Table S1). These findings suggest that variant alleles of *SP1* induce lipid accumulation and inhibit lipophagy in lipid metabolism.

**Figure 1:**
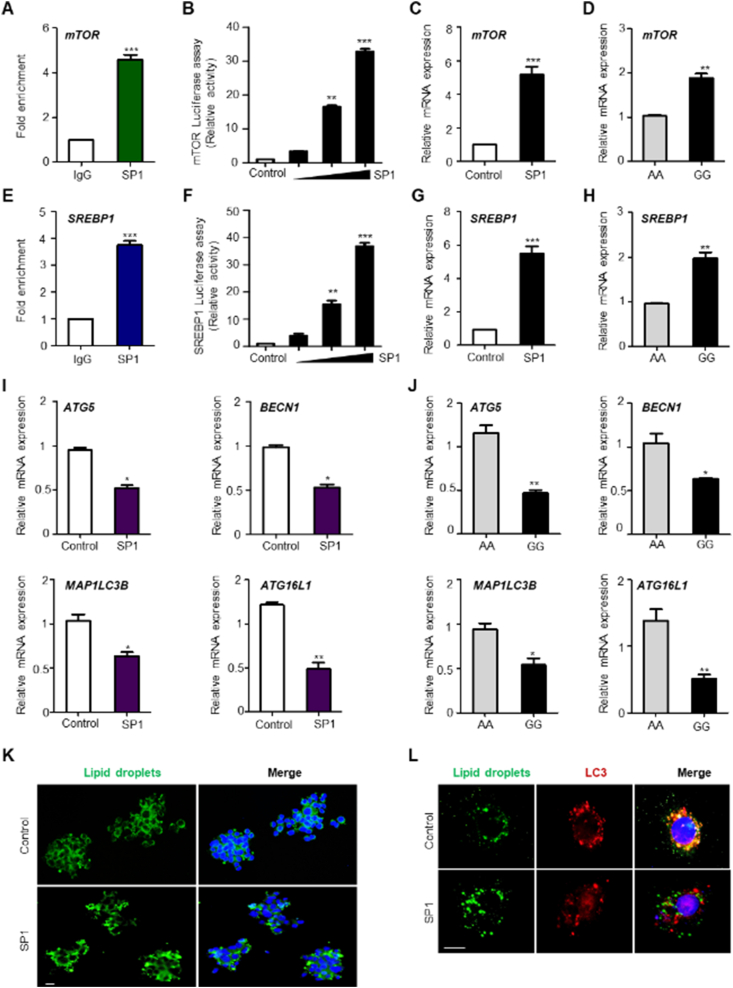
SP1, mediated by gene variants, affects lipogenesis and lipophagy. **(A)** HepG2 cells were transfected with control vector or SP1 constructs and subjected to chromatin immunoprecipitation assays using an IgG antibody or anti-SP1 antibody, followed by RT-PCR to determine *mTOR* expression. **(B)** HepG2 cells were transfected with an mTOR reporter plasmid (1 μg) or the SP1 plasmid (0.1, 0.5, and 1 μg). HepG2 cells were used for luciferase activity assays. **(C)** HepG2 cells were transfected with the SP1 construct (1 μg), and RT-PCR was performed to determine *mTOR* mRNA expression. **(D)** PBMCs bearing the reference alleles or variant alleles of rs11170510 or rs58123204 (*n* = 10, reference alleles AA; *n* = 10, variant alleles GG) were subjected to RT-PCR using a primer for *mTOR*. **(E)** HepG2 cells were transfected with the control vector or SP1 construct (1 μg) and subjected to chromatin immunoprecipitation assays using an IgG antibody or anti-SP1 antibody, and RT-PCR with primers for *SREBP1*. **(F)** HepG2 cells were transfected with an SREBP1 reporter or the SP1 construct (0.1, 0.5, and 1 μg) and subjected to luciferase activity assays. **(G)** HepG2 cells were transfected with the SP1 construct (1 μg), and RT-PCR was performed to detect *SREBP1* mRNA. **(H)** PBMCs were used for RT-PCR for *SREBP1* mRNA (*n* = 10, reference alleles AA; *n* = 10, variant alleles GG). **(I)** HepG2 cells were transfected with *SP1* construct (1 μg), and RT-PCR was performed using primers for *ATG5*, *BECN1*, *ATG16L1*, and *MAP1LC3B*. **(J)** PBMCs were used for RT-PCR of *ATG5*, *BECN1*, *ATG16L1*, and *MAP1LC3B* (*n* = 10, reference alleles AA; *n* = 10, variant alleles GG). **(K, L)** HepG2 cells were transfected with the control vector or *SP1* construct (1 μg). (K) HepG2 cells were stained for BODIPY 493/503. Scale bar: 10 mm. (L) Immunofluorescence microscopy analysis of a merge of lipid droplet and LC3 staining. Scale bar: 10 mm. Experiments were repeated at least three times. Data represent mean ± standard deviation. ∗*P* < 0.05, ∗∗*P* < 0.01, and ∗∗∗*P* < 0.001. PBMC, peripheral blood mononuclear cell; SP1, specificity protein 1; RT-PCR, quantitative real-time reverse-transcription; *mTOR*, mechanistic target of rapamycin; *SREBP1*, sterol regulatory element-binding transcription factor 1; *ATG5*, autophagy-related 5; *BECN1*, beclin 1; *ATG16L1*, autophagy-related 16-like 1; LC3, light chain 3.

Excessive hepatic *de novo* lipogenesis functions as a pathological factor in lipid metabolism and contributes to the development of NAFLD and type 2 diabetes.^5^ In this study, which explored the impact of internal factors on *de novo* lipid metabolism, the *SP1* gene variants up-regulated the transcriptional activity of *mTOR* and *SREBP1* in HepG2 cells. The expression of key enzymes of lipid synthesis, ACACA, SCD, and FASN, was up-regulated in the presence of the variant *SP1* alleles, which increased *SP1* mRNA levels. We revealed a previously unknown role for *SP1* gene variants in contributing to *de novo* lipogenesis in liver cells. The increase in SP1 resulted in a decrease in autophagosomes and autophagolysosomes, and the increased lipid droplets produced by SP1 overexpression were less likely to be removed by lipophagy. These results suggest that an increase in SP1 levels by genetic factors disrupts the homeostasis of lipid droplets. Lipophagy is associated with metabolic diseases, and studies have reported that abnormal control of lipophagy causes obesity, diabetes, alcoholic fatty liver disease, NAFLD, and liver fibrosis. Therefore, investigating the role of genetic factors in lipophagy is important for the prevention and treatment of metabolic diseases. In conclusion, our findings illustrate a previously unrecognized role of genetic variants in *SP1* associated with CS and describe the mechanisms by which it regulates *de novo* lipogenesis through up-regulated transcriptional activation of *mTOR* and *SREBP1* and lipophagy through down-regulated autophagy activation and transcriptional activity of autophagy-related genes in lipid metabolism. This confirms the biological mechanism underlying the clinically known relationship between CS and lipid metabolism through Mendelian randomization analysis of *SP1* genetic variations and experimental validation.

Our study has a few limitations. First, we evaluated whether SP1 functional polymorphisms induce transcriptional activation of mTOR, SREBP1, and autophagy genes; however, we did not examine other genes regulated by SP1. Further, future research is required to identify other genes regulated by SP1 variants. Second, for the same reason, we did not perform Mendelian randomization analysis for all gene variants related to CS. In the future, we aim to confirm the genetic causality linking CS to lipid metabolism parameters. Finally, follow-up data were not available for the KDCC cohort; therefore, we could not detect lipid metabolism-related diseases associated with SP1 variants by analyzing follow-up data in a cohort study. Nevertheless, approaches based on targeting *SP1* genetic variants could provide insights into the diagnosis and prediction of treatment response for diseases associated with abnormal lipid metabolism.

## CRediT authorship contribution statement

**Soo Yeon Kim:** Conceptualization, Formal analysis, Methodology, Writing – original draft, Writing – review & editing, Data curation, Validation, Visualization. **Hyo-Jeong Ban:** Conceptualization, Data curation, Formal analysis, Methodology, Software, Visualization, Writing – original draft, Writing – review & editing, Investigation. **Siwoo Lee:** Data curation, Funding acquisition, Resources. **Hee-Jeong Jin:** Conceptualization, Formal analysis, Investigation, Methodology, Project administration, Resources, Supervision, Visualization, Writing – original draft, Writing – review & editing.

## Ethics declaration

Informed consent was obtained from all participants in the study. The Institutional Review Board at the KIOM and Dunsan Korean Medicine Hospital of Daejeon University reviewed and approved this study (IRB No. DJDSKH-17-BM-12).

## Funding

This study was supported by the “Development of Korean Medicine Original Technology for Preventive Treatment based on Integrative Big Data” grant from the 10.13039/501100003718Korea Institute of Oriental Medicine (No. KSN1739121).

## Conflict of interests

The authors declared no competing interests.

## Figures and Tables

**figs1:**
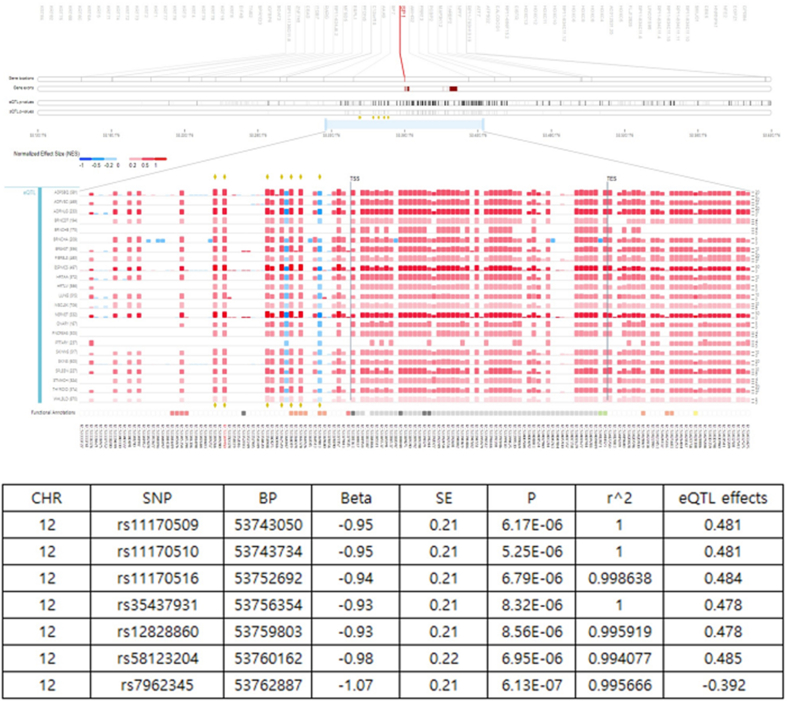


**figs2:**
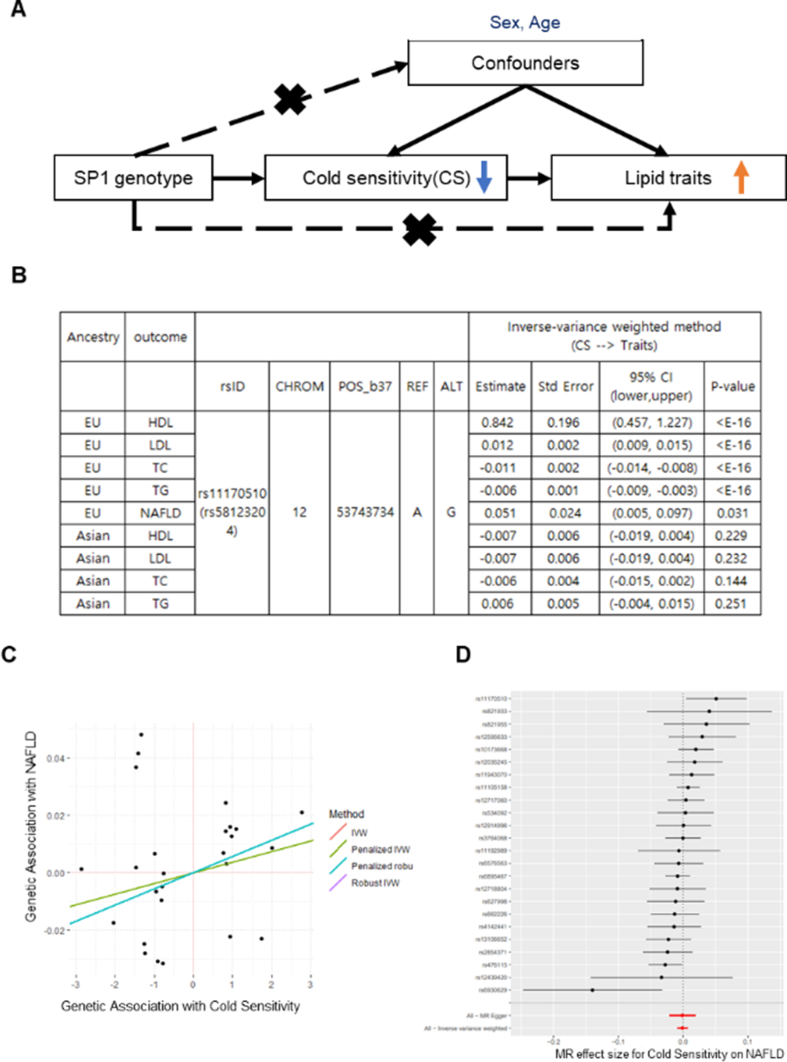


**figs3:**
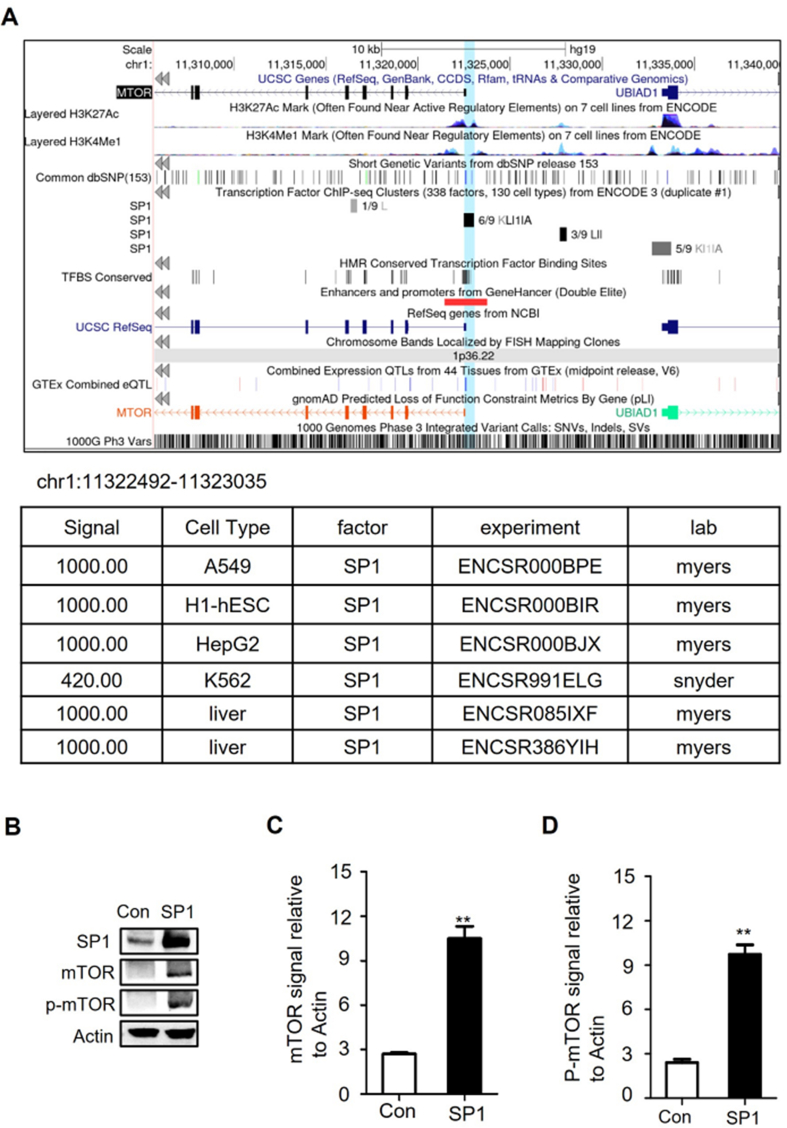


**figs4:**
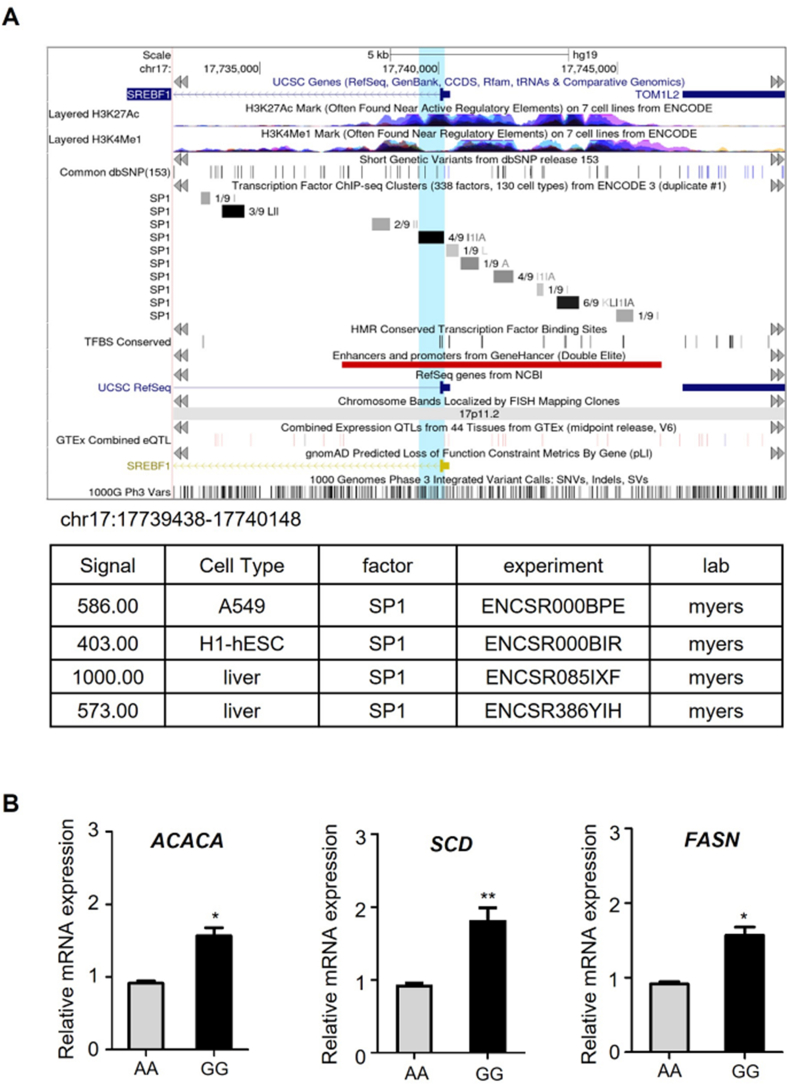


**figs5:**
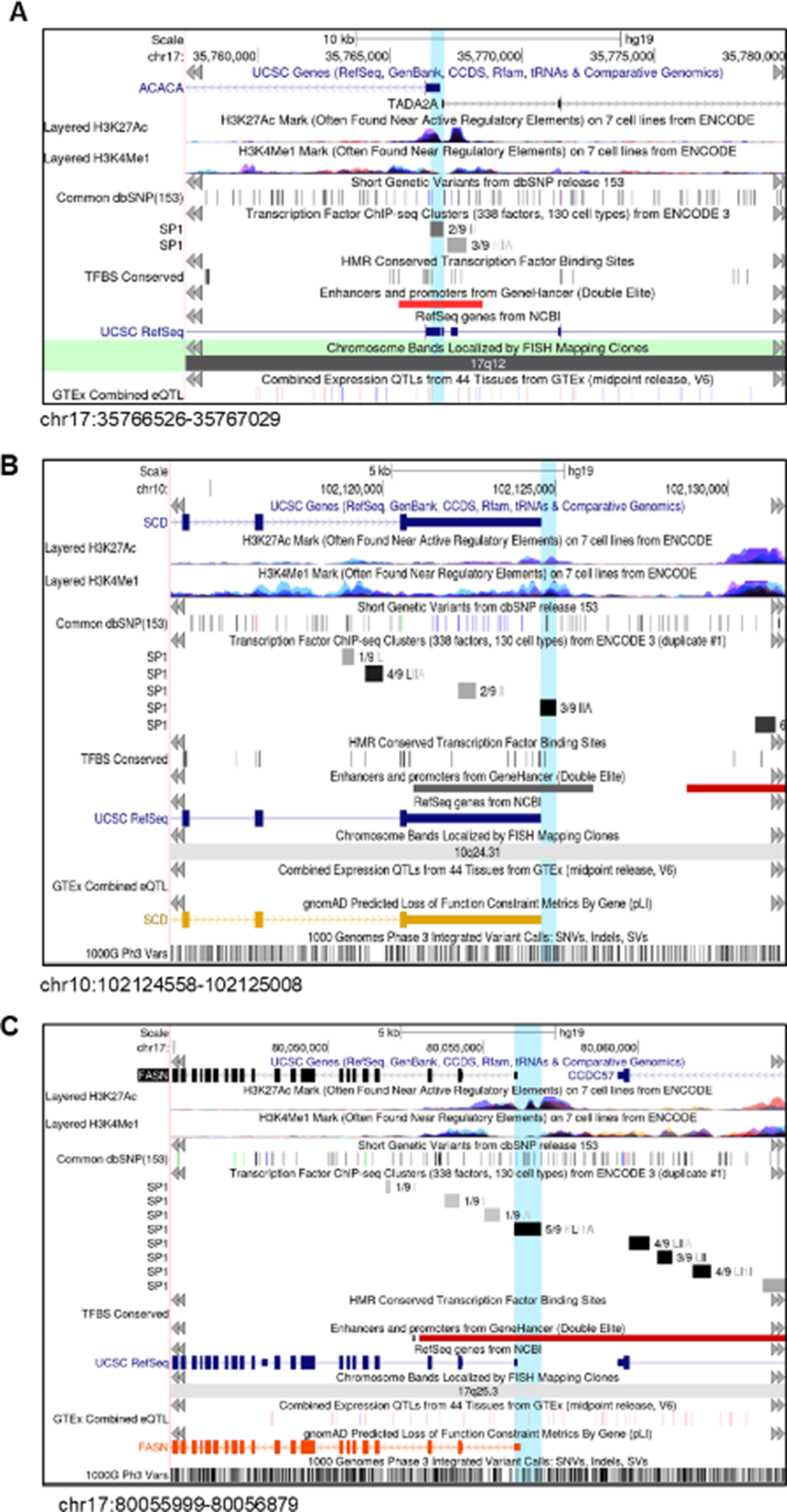


**figs6:**
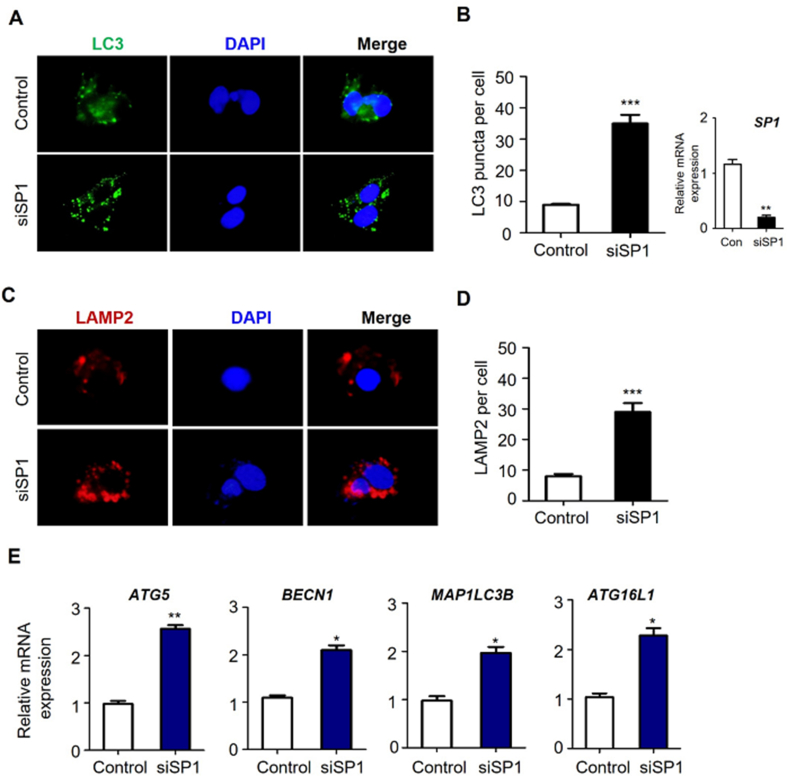


**figs7:**