# Supplementary material for: Regulation of de novo lipogenesis and lipophagy by SP1 gene variants
Source: Genes Dis. 2025 Jun 27;13(1):101722. doi: 10.1016/j.gendis.2025.101722 (PMC12624637; doi:10.1016/j.gendis.2025.101722)
Supplement: Multimedia component 1 [file mmc1.docx]

**Supplementary Information**

This file includes:

Supplementary Background

Supplementary Materials and Methods

Supplementary Results

Supplementary References

Supplementary Figures (S1-7)

Supplementary Tables captions (T1-3)

**Supplementary Background**

All organisms regularly face stress associated with responses to external or internal temperatures. Cold stress, which is a common form of environmental stress, modifies adrenocorticotropic hormones, adrenaline, noradrenaline, cortisol, and glucose levels.^1-4^ Cold hypersensitivity is defined as the experience of a sensation of coldness in an environment that is not considered cold, or a sense of feeling extremely cold when the temperature is only slightly low.^5-12^ Cold hypersensitivity is not only inconvenient to individuals in their daily lives but is also closely associated with diseases, such as allergic rhinitis^6^ and functional dyspepsia.^7^ In particular, cold sensitivity (CS) is known to be associated with lower rates of metabolic and cardiovascular diseases, including dyslipidemia.^8;12^ In these previous studies, total cholesterol (TC) and low density lipoprotein-cholesterol (LDL-C) were reported to be significantly higher in the non-CS group than in the CS group, while high density lipoprotein-cholesterol (HDL-C) was significantly higher in the CS group than in the non-CS group. ^8^

Recently, genetic variations associated with CS have been confirmed using genome-wide association studies (GWAS). Among these variations, the most strongly associated single nucleotide polymorphisms (SNPs) (rs11170510 and rs58123204) showed a strong linkage disequilibrium (r^2^>0.8) with the nearest *SP1* gene.^9^ Specificity protein 1 (SP1), encoded by a gene associated with CS, binds to GC-rich gene promoters as a transcription factor ^13^ and is associated with the lipogenesis pathway in various cells.^14;15^ Lipid biosynthesis, one of the processes involved in lipogenesis, is enhanced by mammalian target of rapamycin (mTOR) complex 1 (mTORC1)-mediated activation of the transcription factor sterol regulatory element binding protein 1 (SREBP1).^16^ SREBP1 contributes to the expression of critical enzymes in lipid biosynthesis, including fatty acid synthase (FASN), acetyl-CoA carboxylase alpha (ACACA), and stearoyl-CoA desaturase (SCD).^17^ The mTOR/SREBP1 pathway is critical in lipid synthesis, and the transcriptional activity of *mTOR* and *SREBP1* is regulated by SP1.^18^

Autophagy contributes to intracellular homeostasis by damaging organelles, longevity proteins, and aggregates that break down within intracellular lysosomes. Lipophagy, which is a selective form of autophagy, is a necessary mechanism for targeting lipid droplets and maintaining lipid homeostasis.^19;20^ Lipophagy is involved in metabolic disorders such as obesity, diabetes mellitus, alcoholic fatty liver disorder (AFLD), nonalcoholic fatty liver disease (NAFLD), and liver fibrosis.^21-25^ SP1 is also known to be involved in the autophagy mechanism .^26-29^

GWASs using large-scale data from the Million Veteran Program,^30^ UK Biobank,^31^ and the BioBank Japan Project ^32^ have been conducted to identify genetic factors associated with blood lipid level variations, and each of these studies have identified thousands to tens of thousands of genet variants.^33-37^ However, as a phenotype is indirectly influenced by more genes than the number that directly and largely affect the phenotype,^38;39^ it is still difficult to identify all of genes affecting lipid metabolism through GWAS.

**Supplementary Materials and Methods**

**Data**

In this study, we conducted Mendelian randomization (MR) analysis using publicly available GWAS data to investigate the influence of genetic variations in SP1, a CS-associated gene, on lipid metabolism. The ongoing Korean Medicine Daejeon Citizen Cohort (KDCC) cohort study ^33,40^ assesses associations between lifestyle factors and chronic diseases, with recent publication of GWAS results related to CS.^9^ We utilized GWAS summary data for lipid traits ^32;34;41^ (LDL-C, HDL-C, TGs, and TC) and NAFLD ^42^ from prominent databases such as GWAS catalog ^43^ and OpenGWAS.^44^

**Genetic instruments and MR**

In epidemiology, MR involves using genetic variation as an instrumental variable (IV) in nonexperimental designs to establish causality for modifiable exposures on outcomes.^45^ In principle, all common SNPs can be employed as IVs in MR analysis; however, GWAS results are initially clumped to identify only the most significant SNPs within Linkage Disequilibrium (LD) blocks. This is done to preserve SNPs with the strongest statistical evidence while reducing the correlation between the remaining SNPs. From the GWAS results for CS, nine SNPs obtained through clumping (using the settings ‘-clump-p1 0.00005 –clump-r2 0.5 –clump-kb 250’ in plink 1.90 ^46^) were selected as IVs. Confounding factors, such as sex and age, which could confound CS, were adjusted for during the GWAS analysis (Fig. S1B). To investigate the causal relationships between CS and lipid metabolism, we constructed a publicly available dataset ^32;34;41;42^ related to lipid metabolism. To account for differences among individuals and races, we used the results of research on Europeans and Asians from a cohort that was not used for our CS-GWAS analysis. Analysis was conducted by extracting IVs from GWAS results for representative lipid metabolism variables (LDL-C, HDL-C, TG, and TC), and for NAFLD arising from issues with lipid metabolism. The analysis was performed using Mendelian Randomization ^47^ package in R version 4.1.3. We employed inverse-variance weighting methods to evaluate the causal effects of CS-related gene variants. This method calculates the IVs using generalized weighted linear regression when the IVs are correlated.

**Cell culture**

HepG2 cells (HB-8065) were purchased from the American Type Culture Collection (Manassas, VA, USA) and grown in Dulbecco’s modified Eagle’s medium (Lonza Bioscience Solutions, Basel, Switzerland) containing 10% fetal bovine serum, and 1% antibiotics in a conditioned environment at 5% CO_2_ at 37 °C.

**Plasmid construction**

The *mTOR* and *SREBP1* promoter plasmids were generated by Cosmogeneth (Seoul, South Korea). The *mTOR* promoter or *SREBP*1 promoter was integrated into the pGL4.23 vector (E841A; Promega, Madison, WI, USA). The SP1 construct (24543) was obtained from Addgene (Watertown, MA, USA). Transcription factor sites in the promoter regions of *mTOR* and *SREBP1* were confirmed using a bioinformatics tool (<http://www.cbrc.jp/research/db/TFSEARCH.html>).

**Luciferase assays**

HepG2 cells (5 × 10^5^ cells per well) were transiently co-transfected with the pGL4.23 [luc2/minP] vector (0.5 µg) (E841A, Promega), pGL4.23-*mTOR* promoter (0.5 µg), pGL4.23-*SREBP1* promoter (0.5 µg), or SP1 construct (0.1, 0.5, or 1 µg), and pRL vector (Renilla luciferase gene plasmid) (10 ng), using Lipofectamine 3000 (11668027, Invitrogen, Waltham, MA, USA). The cells were cultured in 12-well plates for 24 h. Luciferase activity assays were performed using the Dual-Luciferase® Reporter Assay System (E1910, Promega). Luciferase activity was normalized to that of Renilla luciferase. The *mTOR* promoter site was -2940/+0. The *mTOR* promoter sequences were used: 5′- agtgaaaatgtaaaatttagaggaatacttatctcacaggacaatgaaaagaggctaaatctaaagtagccattattactcgagaggctgaggcaagagaatcgcttaaacctgggaggcagaggttgcagtgaggtgagatggctcccctgcactccagcctgggtgacagagtaagattctgtctcaaaaaaaaggagcaattatatatatatatatatttatatatatatatatataattttttttttttgagacagtgtctcactctgtcaactcaggatggaatgcagtggtgcagtggtgagatcacagctcactgcagccttgacctcctgggctcaagtgatcctcccacctcaacctcttaagtagctgggaccacaggtgcatgccaccacaccaactaattttttgcattttttgtagagatgaggtttcaccatgttgcccaggtgggctcaaacaatccacctgcctcagcctcccaaagtgttgggtcactgtgcctggccaatatcatagatattaacaataacaacaacaacaacaaaaaccctgccaaacaaagcattttgttagccacggaatattgacatctttatttgtttatttatttatttatttgagatggagtctcactatgttgcccaggctggtctcaaatgcctgggctcatgcaatcttcctgctttggcctcccaaagtgctgggattacaggtgtgagccaccgcacccagcccaactatctttaatttggcagtttagaccttctgaaaactcatttcttctaccttcacttgtttttattacataagtaattgataatgtataaagaaaaaaagtgtgtctttcccaaattacatcttagttgtgcacatttcccccatgtaaacagagacatataagaaatgcataaatatttttgtaaatggttgtattatacttacattgttgcacaacttgtaatttttaaactagttgtgaaccttttccttcttagcaaatattcatctaattaccactatttattgttattattattatttgagacaggatctttctctgctgcccaggctgcagtgcagtggagatcacagatgactgcaaccttgaagtcatagactcaagtgattctcgtgcctcagccttccaagtagctgggactacaggcatgtgctaccacacacagctaatttttaaatttcttttgtagagacaagggtctcactatgttgctcaggctggtctggaactcctggcctcaagcaatcctgcctcggcctcccaaagtgctgggattccagggatgagccaccatgctaggctctaaattataattattaatggctatgtatttttcttattatgaatgcatctttttttttttttttttttttttttgagatggagtctcgcactgtcaccccagctggagcgcagtggcgcgatctcggctcactgcaacctccgcctgctggcttcaagggattttcctatctcagcctcctgagtagctggcattacaggcacctgccaccacgcctacctttttttttttttttttgtatttttagtaaagacggggtttcactatgttggtcaagctggtctcaaactcctgacctcatgatccgcccgcctcggcctcccaaagtgctgggattacaggtgtgagccaccgcgcccaaacgaatgcatcatattttattggactcctactttgaaatatttaggaattttcagtgtttactatgacaaatagcactgtgattaaaaaaaaatgtagtcaaatccacttttgctgttgatttttttttttttaatctgtagagacatcgtctcgctatgttgcccaggctggtctggaactcctggcctcaagcgatcctcctgctttggcctcccaaagtgcgtgggccaccgtgaccagcagttatttattttctttggttgtgtgtgtgtgtgtgtgtgtgtg

tgtgtgtgtgtgtgtgtatgtatgtattgagacggagcttgctctgttgcccaggctagagtgcagtggcacgatctcggtgcatgcaaccttcacctcccgggttcaggcgatactcgtgcctcagcctcccgagtatctgggattacaggcgtgagccaccatgcccagctaatttttgtattttcagtagagacggggggtgcgggtgggggaatttcaccatgttgaccaggctggtctcgactcctgacctacggtgatccgcctgcctcggtctcccaaagtgctggaattatgggcgtgagccaccgcgccgggccagctattgattttttgaaaggatttttaaaaaataatgttgctttgaaaatttttatttatgactagctcttttggttaaaacttggctatggtttctctggagtgatcctgagttggataattttgggaaagttagagaatctaattaaaaacattttcaaatgcttattaattgtaacaaaagagatatcacaggatccataaagagcgctagcccgaaacgtcttttgatgcagtaattcctgggcaccggcatcgctcacattctcctctcagaattacaccgggaggggtcgcgaacctctgctagccgctctcgctgcgatccggagatacccagacaccggactccttgagttcacagcccggatgcagaagggaatcctagcagcgccgtaccggatgtgtgagtggaagtgactgaggtgaactcacgaccgattggttctcccgagtggacccaagcctgttttcacctgccactggccgcgtggtttgtctatttgaacagtccccgccccctggagaagccgggtcctgaagcttctcttcttcattactattggcctgtttttcagtccatcttctccctatacctgtcgattggtcctcagggctgggaaccctccttccctc -3′ The *SREBP1* promoter site was -3660/+0. The *SREBP1* promoter sequences were used: : 5′- gctggacaggacaagcgtcctggaagaggccctgcccgtgctggacgcctgcccgaaaaggctcgaaacccacagaagctgcctctcagtctctaagaggcttggagaggaagcggggagatgcgaattcctatctcccagttggcaacgccgaggtcgggcaggggccgggctgggtgacctggaaggaatcctccgctctgggcgcgccacgcagtcccgggtggggctgtcccgtgttagcccttccggtgcccgggacgcgcacctggcggcattcctggccaggtgtctggactgggggctgagcccagcctgtccccgccgcccctccctacctcccgggtagagcgggcgcggcgcatgtgacccagggctgggctcccgggagttacgcgctgacgccgcgtcaccccactccgggccgggcgcccattggctgcgccgggcccgcgggggcggggctggtctggctctgcgccccggctcccctgggtctccagccgctgccctggcccgcgcgcgtgcggagccgccccggctctccggctacctccagtccagacaaaaccaggggcagcagtgctgtgaggtcctgagcaagtcgcttcaccgtcccgctccacgtgcctcaatttactcatctgtaaaatgggatgg

taacagcctcaaccgcctgaggctgtcgagaggagtgaatgggtttaaaccgcactgaacactctgtaagcgctcagcaagtaaactgtgccgaacctgcccgccggggtcaccgtggaccaggctggatccctgacccctgataggcacaccattacagaggggcttgtgtccacgttccttgggcgtgctcaacgctccccagccaaccggggcccgagggtcttgtttggaggtctcaggatctttgaggagagaaactgccaagacaagcatgttccccctgaaaaatggatcccctcttctgttttcccctaccctcacgttgagggttgccctggtaatcccaggctgtggggcaaagatttgtttctttggtggcaaagatgtaaattcttcccacccccacgttggactgtgccccatgggggttggattttctggggtgcaggtcttctgttgaccttgtcttaccttctttccctttcccctaactcctgcagttatggagtgaatatttattgagctattctatttacatatgtggtaacccatttaaaaacggtgaggcgggttgggtgcagtagctcacgtctgtaatccgagcactttgggaagccgaagtgggtagattactggaggctaggagttcgaaaccagtctgaccaacatgacaaaaccccatctctactaaaaatacaaaaattagccgggcgtggtagtgcatgactgtaatcccatctactcgggaggctgagggaaaagaaatgcttgaacccgggaggccgacgttgcagtgagccaagattgtgccactgcactccagcctgggcaacagagcgagactctgtcttaaaaacaacaacaacaacaacaacaacaacaacaaaaacagtgatagccaggtgcagtgactcacacttgcaatcccagcactttgggggggccgaggtaggtggatcacctaaggtcaggagttcaagaccagcctggccaaaatggtgaaacctcatctctactaaaaatacaaaaattagcctggcatggtggcgtgcgcctgtaatcccagctactcaggaggctgagacagaattgcttgaacctgggaggtggaggtcgcagtgagccgagatcatgccgctgcactccagcctgggtgacagagagagactttgtctcaaaacaaaaaacaaaacaaaacaaaacaaaaaacagtgaggcagatctgttgtgatgatgactccaaaacaccctccctgcctctgcagagggactgcagaaagggttattcccattttatagatacagtagctgagactcagaagtgaggtattggatccaggtcacacagcaagcaggtgaaaacccagatcacctgcctagctctgaagaaaatggtatttaggcttcacccagcacttcctatcccaccccctccctgggaagggctgtttaaattttcagggaagtcaccagcttcctgcagcctctagagtgttggtgggggtggggcactgaggagaagccagctttgttctctgtgttctccagcagttgttcatctggagggagtgggtcctgggtggacccttgagcagggctacttggggagatgtggtttggcgacccctatgacttctggcgccgctattctgggtaattttccaccgcagccacttctgggagaggaacaaagggagctggatgtccaggctgagccccagggacttgggctctgtggcttctctcccccacacaccccttctaaaatgcatcatgaatgttactcctgcttagggcgtggccagataggctatatctggagtttgagcaaggcagtctgcaggatggcttgacttatgaaggtctggggtcgggaaggcctgaggcccaggcccctgatgagtttcctggactgccctccaccaagggtgcttctctctgcttgcatacctctggggacagggagctcactgccttatcacattcagattcctgaatgttaattctggtttatttcaacccacctcattgggaccccttccctccttcctgccccacctggctctgtccctaggccacagaaccaggttcggtttccagccctcttctcaacagggctgcctgctctgatctagtcccagcttgtgatgatccagggcagcctggctctgatctaaagcacagctacctcttccttgcggcccctatcctggctgctcctgggaataagtgccaaatctggggtcagacagccctggggccagtcttccttgggtactggcttcctccttcaggagctgcactgggcccactggtatcctatccctacagctggatctgggaggaaaccagatgacgaaattccagcctctttctttggccactcctgtcctcaagaggccaatcttctggtttctttgcagagagggggcaggctgatctcacaggtcatgctcccctccacattgtcactagcctcccagcctgcccgtgagaaagcatcattaggcccatgttacaaatgaggaaaattgaggcagagtgatgtaactggcccagcagttacatcaggcctgctcacaacacagcaggcctgggacccctataacttggatcctggtctgtcttgttctaaagagtcaaatctaggaaatgaggaaatgaagtttgggatgggcccaggcctggggcttccactcggcttccttgcttggtgctggagaaacagaggcccagagagggggctcggcttgcccgcgttcccgcagcagccggccagaggccgctgccattgtgcgcgaggctggataaaatgaatgactggagggcgctctggaggaggggccggctgaggggagatttgtggcgcagaccggggatcaggggtcccccgctctctcaaggtggggcggggccgtctatctgggagggcgggtcctccccgaaaggccccgcctccgcctcgaccgcccagcagagctgcggccgggggaaccc-3′

**Real-time qPCR**

A RNeasy Kit (74034; Qiagen) was used to extract the total RNA of PBMCs (1 × 10^6^). One microgram of RNA was transcribed into cDNA using the SuperScript Synthesis System (11904018, Invitrogen). cDNA (10 ng) was amplified using primers and the SYBR Green PCR Master Mix (4367659, Applied Biosystems, Waltham, MA, USA) in a Rotor-Gene Q 2plex system (9001620; Qiagen, Hilden, Germany). Real-time PCR was performed using the following thermal profile: 40 cycles at 95 °C for 30 s, 53 °C for 30 s, and 72 °C for 1 min. We derived quantitative reverse-transcription (RT)-PCR data using the 2^ΔΔCt^ method, with *GAPDH* as the internal control gene; the results are expressed as relative fold-changes. The following primer sequences were used: *mTOR*, 5′-attggaagaatcaaagagca-3′ (sense) and 5′-gttgccaggacattattgat-3′ (antisense); *SREBP1*, 5′-catgcttcagcttatcaaca-3′ (sense) and 5′-tcaagagaggagctcaatgt-3′ (antisense); *ACACA*, 5′-gcacagttcagttcctcttc-3′ (sense) and 5′-caacaactcccacaggtatt-3′ (antisense); *FASN* 5′-gtggacctgatcatcaagag-3′ (sense) and 5′-catacctgggagaggttgta-3′ (antisense); *SCD*, 5-tacggctctttctgatcatt-3′ (sense) and 5′-tacctcctctggaacatcac-3′ (antisense); *ATG5*, 5′-acaaagatgtgcttcgagat-3′ (sense) and 5′-gataatgccatttcagtggt-3′ (antisense); *BECN1*, 5′-aggatgatgtccacagaaag-3′ (sense) and 5′-gtgtccagctggtctaaaag-3′ (antisense); *ATG16L1*, 5′-caaatgctatcaagcagaca-3′ (sense) and 5′-aagaaccttttccactttcc-3′ (antisense); *MAP1LC3A*, 5′-ctggacaagaccaagttttt-3′ (sense) and 5′-tttctcctgctcgtagatgt-3′ (antisense), and *GAPDH*, 5′-aacctgccaaatatgatgaca-3′ (sense) and 5′-ataccaggaaatgagcttgac-3′ (antisense).

**Chromatin Immunoprecipitation (ChIP)**

ChIP was conducted using the SimpleChIP Chromatin IP Kit (17-1169486**,** Cell Signaling Technology, Danvers, MA, USA). HepG2 cells (1 × 10^7^) were plated in 100 mm dishes until 70–80% confluency and were then fixed with 37.5% formaldehyde (1% final concentration; F8775, Sigma-Aldrich, St Louis, MO, USA). The cells were washed and collected in 1× phosphate-buffered saline (PBS; 10010023, Gibco, Billings, MT, USA). Cross-linked chromatin was cut to approximately 150–500 bp using micrococcal nuclease. The DNA–protein complexes were immunoprecipitated using a mouse monoclonal anti-SP1 antibody (sc-17824, Santa Cruz Biotechnology, Dallas, TX, USA) and an anti-mouse IgG control antibody (sc-17824, Santa Cruz Biotechnology). After DNA recovery, the enrichment of SP1 in mTOR or SREBP1 was assessed via RT-PCR. ChIP primer site for *mTOR* was -840/-820 and that for the mTOR promoter was -680/-660. ChIP primer sequences used were: *mTOR*, 5′- gcctcagcctcccgagtatc -3′ (sense) and 5′- cataattccagcatttggg-3′ (antisense). ChIP primer site for *SREBP1* was -180/-160 and that for the *SREBP1* promoter was -20/-1. ChIP primer sequences used were: *SREBP1*, 5′- tgactggagggcgctctgga -3′ (sense) and 5′- gggttcccccggccgcagct-3′ (antisense).

**Immunofluorescence**

HepG2 cells (5 × 10^5^ cells per well) were seeded in 12-well culture plates on cover glass and cultured for 24 h and then transfected with the SP1 construct (1 µg) for another 24 h. Cells were washed with 1× PBS and stained using BODIPY 493/503 (17-1084672, Invitrogen) in a culture incubator at 37 °C for 15 min. HepG2 cells (5 × 10^5^ cells per well) were seeded in 12-well culture plates on cover glass for 24 h. siSP1 (sc-29487-V, Santa Cruz Biotechnology) was transfected into these HepG2 cells using Lipofectamine RNAiMAX (13778150, Invitrogen) for 24 h. These cells were then fixed with 4% paraformaldehyde for 10 min and subsequently permeabilized with 0.25% Triton X-100 for 10 min. The cells were then incubated with the anti-LC3 (PM036, MBL) or anti-LAMP2 (sc-18822, Santa Cruz Biotechnology) antibodies for 16 h at 4 °C. The primary antibodies were removed, and the cells were stained with a secondary antibody conjugated to Alexa Fluor 488 (A-11008; Molecular Probes, Eugene, OR, USA) or Alexa Fluor 594 (Molecular Probes, A11007) for 2 h at room temperature. The secondary antibodies were removed, and the cells were washed with 1× PBS. The cells were then incubated with DAPI (D8417, Sigma-Aldrich) for 5 min at room temperature. Images were examined using a Zeiss LSM 710 confocal microscope (Carl Zeiss, Oberkochen, Germany). Images were obtained using ZEN 2009 software (version 5.5 SP1; Zeiss). Each experiment was conducted on triplicate coverslips. The results are presented as the average and standard deviation. ImageJ was used to quantify the image data.

**RNA-seq data and clinical information analysis**

To analyze clinical information regarding lipid metabolism based on the genotypes of *SP1*, we utilized clinical data, including age, sex, height, and weight, and LDL-C, HDL-C, TG, and TG levels, from KDCC participants. Variant alleles of the functional SNPs of *SP1,* including rs11170510 and rs58123204, were carried by 118 individuals. Additionally, we performed RNA-seq analysis using PBMCs obtained from 100 participants to determine the changes in the expression of genes due to SP1 variants. The data were obtained from the Gene Expression Omnibus database (accession number GSE200744) ^48^ and were analyzed using R program (version 4.2.2). For the key genes within the lipogenesis and autophagy pathways, we examined the read counts of individuals with the variant alleles types (GG) of *SP1* among the 100 participants.

**Western blotting**

HepG2 cells (1 × 10^6^ cells per well) were transiently transfected with the SP1 construct (1 µg) using Lipofectamine 3000 and were cultured in 12-well plates for 24 h. Cells were lysed using RIPA lysis buffer containing PI cocktail (Roche, REF11697498001). The cell extracts were boiled in SDS sample buffer at 95 °C for 10 min. Total protein was separated using 10% SDS-polyacrylamide gel electrophoresis and then transferred to polyvinylidene difluoride membranes (Bio rad, BR1620177). Membranes were blocked in 5% bovine serum albumin in in Tris-buffered saline containing 0.1% Tween 20 (TBS-T) for 1 h. Membranes were incubated with primary specific antibodies against SP1 (sc-17824, Santa Cruz Biotechnology; 1:1,000), mTOR (2972S, Cell Signaling Technology, 1:1,000), phospho-mTOR (2971S, Cell Signaling Technology, 1:1,000), SREBP1 (ab28481, Abcam, 1:1,000), or anti-β-actin (sc-517582, Santa Cruz Biotechnology, 1:1,000) overnight at 4 °C and were then incubated with appropriate secondary antibodies at room temperature for 1 h. Antibody-binding proteins were detected by chemiluminescence using an ECL reagent (17-1084359; Thermo Fisher Scientific). Bands were developed using a Vilber chemiluminescence analyzer (Vilber Lourmat, Fusion SL 3, Eberhardzell, Germany). The antibody-binding bands were analyzed for densitometry using ImageJ.

**Statistical analysis**

The results are presented as the mean ± standard deviation (SD). The statistical significance of the difference between paired groups was analyzed using Mann–Whitney *U* tests in SPSS Statistics version 21.0 (IBM, Armonk, NY, USA) for Windows.

**Supplementary Results**

**Gene variants of *SP1* strongly influence lipid levels**

Numerous GWASs of lipid metabolism have been conducted; however, some key genetic factors have not been identified. Through MR analysis of SP1 genetic variations, our study established a causal link between decreased CS, driven by specific genetic factors, and changes in lipid levels (TC, LDL-C, TG, and HDL-C levels). The GWAS results revealed that CS-associated genetic loci were distributed across a broad region of the chromosome; however, nine crucial SNPs were identified after excluding correlated SNPs based on LD. The SP1-associated genetic region contained seven SNPs that exhibited strongly correlated LD values (*r^2^* > 0.99; Fig. S1). Subsequently, we examined the influence of the regulating tagSNP (rs11170510) of SP1 on lipid metabolism. Causal analysis, with inverse-variance weighting, was performed using independent GWAS results related to lipid metabolism obtained from European and Asian cohorts. The results revealed a significant causal relationship between lipid metabolism and CS for Europeans; however, no significant effect was observed for Asians (Fig. S2B). These findings suggest a causal relationship between genetic inhibition of CS and altered lipid levels. While MR is a useful tool for inferring causal relationships between exposures and outcomes, it can also misidentify causal relationships in the presence of small genetic influences. Therefore, we conducted experiments to verify the effect of gene variants of SP1 on lipid metabolism.

**Gene variants of *SP1* regulate mTOR and SREBP1 involved in de novo lipogenesis**

As shown in Figure S1 and 2, SP1, along with its functional SNPs, is associated with lipid metabolism. SP1 acts as a transcription factor in various cellular signaling pathways.^49^ Thus, we used the ENCODE (https://www.encodeproject.org/) tools to find the difference in transcriptional regulation of the promoters of various genes associated with lipid metabolism that are regulated by SP1. We found that among these genes, *the* transcriptional activity of *mTOR* was increased (Fig. S3A). An analysis of ENCODE ChIP-seq data revealed a high-motif consensus sequence near the *mTOR* promoter region (chr1:11322492-11323035). Furthermore, the presence of H3K4Me1 and H3K27Ac epigenetic markers in this region suggested their involvement in transcriptional activation (Fig. S3A). It was confirmed that transcription activity of mTOR was regulated by SP1. Subsequently, we determined whether the interaction between SP1 and the *mTOR* promoter region was correlated with promoter activity. Luciferase assays were performed using 3-kb DNA promoter constructs of *mTOR* and SP1 construct. It was confirmed that SP1 increased mRNA expression of *mTOR*. In addition, western blotting was conducted to confirm the effect of SP1 on mTOR protein levels in HepG2 cells. SP1 overexpression upregulated the total protein and phosphorylation of mTOR (Fig. S3B, C, and D). These results demonstrated that the direct interaction of SP1 with the *mTOR* promoter site enhanced the transcriptional and translational activation of *mTOR*. We previously reported that *SP1* harbored functional SNPs, in which the reference allele was AA, and the variant allele was GG for rs11170510 and rs58123204, respectively. The presence of the variant allele was associated with high *SP1* expression.^9^ Thus, we considered whether the enhanced expression of SP1 because of the GG variant alleles at rs11170510 and rs58123204 would influence mTOR expression in the peripheral blood mononuclear cells (PBMCs) of the KDCC cohort. It was confirmed that mTOR expression was high in the GG variant alleles of SP1. We investigated whether SREBP1 is concurrently regulated by SP1 in the mTOR-SREBP1 signaling pathway during de novo lipogenesis. In the ENCODE ChIP-seq data, the strongest SP1 target peak cluster (chr17:17739438-17740148) was identified within intron 1 of SREBP1. An enrichment of histone markers (H3K4me1 and H3K27Ac) was observed around the SP1 binding site. Using in silico data analysis, we found that SP1 showed high occupancy of the promoter region of *SREBP1* (Fig. S4A). Several studies have shown that SREBP1 is an important transcriptional regulator of lipogenesis.^50^ We examined whether ACACA, SCD, and FASN levels, regulated by the SREBP1 transcription factor during lipogenesis, were affected by gene variants of SP1 using human PBMCs. RT-PCR revealed that the mRNA levels of *ACACA*, *SCD*, and *FASN* were enriched in PBMCs harboring *SP1* variant alleles. We confirmed that the gene variants of *SP1* influenced the mRNA levels of *ACACA,* *SCD,* and *FASN* in human PBMCs. ChIP-seq analysis using ENCODE confirmed substantial SP1-binding in the promoter regions of *ACACA*, *SCD*, and *FASN* (Fig. S5A, B, and C). In addition, using RNA-seq analysis, the expression of genes involved in the lipogenic pathways was confirmed to be high in PBMC samples (n = 100) bearing variant alleles (Table S2) in the KDCC cohort. ^40;51^ These results suggest that the lipogenic pathway genes are regulated by the gene variants of *SP1*.

**Genetic variants of *SP1* inhibit autophagy**

Using RT-PCR, we examined whether SP1 regulated autophagy-related genes. The mRNA levels of autophagy-related genes, *ATG5*, *BECN1*, *ATG16L1*, and *MAP1LC3B*, were significantly upregulated in si*SP1* HepG2 cells compared with those in control cells (Fig. S6E). Using RNA-seq analysis of human PBMCs in the KDCC cohort, we confirmed that human PBMCs bearing variant SP1 alleles showed low expression of autophagy-related genes (Table S3). These results suggest that *SP1* is associated with downregulated autophagy activation and transcriptional activity of autophagy-related genes according to the variant alleles.

**REFERENCE**

1. Gein SV, Sharav’eva IL. Immunomodulating Effects of Cold Stress. *Biology Bulletin Reviews.* 2018;8(6):482-488.

2. Cong P, Liu Y, Liu N, et al. Cold exposure induced oxidative stress and apoptosis in the myocardium by inhibiting the Nrf2-Keap1 signaling pathway. *BMC Cardiovasc Disord.* 2018;18(1):36.

3. Blondin DP, Labbe SM, Phoenix S, et al. Contributions of white and brown adipose tissues and skeletal muscles to acute cold-induced metabolic responses in healthy men. *J Physiol.* 2015;593(3):701-714.

4. Jurado-Fasoli L, Sanchez-Delgado G, Di X, et al. Cold-induced changes in plasma signaling lipids are associated with a healthier cardiometabolic profile independently of brown adipose tissue. *Cell Rep Med.* 2024;5(2):101387.

5. Hur YM, Chae JH, Chung KW, et al. Feeling of cold hands and feet is a highly heritable phenotype. *Twin Res Hum Genet.* 2012;15(2):166-169.

6. Yen HR, Liang KL, Huang TP, Fan JY, Chang TT, Sun MF. Characteristics of traditional Chinese medicine use for children with allergic rhinitis: a nationwide population-based study. *Int J Pediatr Otorhinolaryngol.* 2015;79(4):591-597.

7. Bae KH, Lee JA, Park KH, Yoo JH, Lee Y, Lee S. Cold Hypersensitivity in the Hands and Feet May Be Associated with Functional Dyspepsia: Results of a Multicenter Survey Study. *Evid Based Complement Alternat Med.* 2016;2016:8948690.

8. Bae KH, Go HY, Park KH, Ahn I, Yoon Y, Lee S. The association between cold hypersensitivity in the hands and feet and chronic disease: results of a multicentre study. *BMC Complement Altern Med.* 2018;18(1):40.

9. Kim SY, Ban HJ, Lee S, Jin HJ. Regulation of CIRP by genetic factors of SP1 related to cold sensitivity. *Front Immunol.* 2022;13:994699.

10. Yokoyama K, Yamada Y, Akamatsu Y, et al. Effects of Capsinoids on Daily Physical Activity, Body Composition and Cold Hypersensitivity in Middle-Aged and Older Adults: A Randomized Study. *Nutrients.* 2020;12(1).

11. Bae KH, Lee Y, Go HY, Kim SJ, Lee SW. The Relationship between Cold Hypersensitivity in the Hands and Feet and Health-Related Quality of Life in Koreans: A Nationwide Population Survey. *Evid Based Complement Alternat Med.* 2019;2019:6217036.

12. Park AY, Cha S. Effects of cold sensitivity in the extremities on circulating adiponectin levels and metabolic syndrome in women. *BMC Complement Altern Med.* 2017;17(1):150.

13. Imanishi M, Imamura C, Higashi C, et al. Zinc finger-zinc finger interaction between the transcription factors, GATA-1 and Sp1. *Biochem Biophys Res Commun.* 2010;400(4):625-630.

14. Cheng Y, Huang L, Ping J, Chen T, Chen J. MicroRNA-199a-3p attenuates hepatic lipogenesis by targeting Sp1. *Am J Transl Res.* 2017;9(4):1905-1913.

15. Lu S, Archer MC. Sp1 coordinately regulates de novo lipogenesis and proliferation in cancer cells. *Int J Cancer.* 2010;126(2):416-425.

16. Bakan I, Laplante M. Connecting mTORC1 signaling to SREBP-1 activation. *Curr Opin Lipidol.* 2012;23(3):226-234.

17. Chu K, Zhao N, Hu X, et al. LncNONMMUG027912 alleviates lipid accumulation through AMPKalpha/mTOR/SREBP1C axis in nonalcoholic fatty liver. *Biochem Biophys Res Commun.* 2022;618:8-14.

18. Ravi V, Jain A, Khan D, et al. SIRT6 transcriptionally regulates global protein synthesis through transcription factor Sp1 independent of its deacetylase activity. *Nucleic Acids Res.* 2019;47(17):9115-9131.

19. Singh R, Cuervo AM. Lipophagy: connecting autophagy and lipid metabolism. *Int J Cell Biol.* 2012;2012:282041.

20. Ward C, Martinez-Lopez N, Otten EG, et al. Autophagy, lipophagy and lysosomal lipid storage disorders. *Biochim Biophys Acta.* 2016;1861(4):269-284.

21. Byun S, Seok S, Kim YC, et al. Fasting-induced FGF21 signaling activates hepatic autophagy and lipid degradation via JMJD3 histone demethylase. *Nat Commun.* 2020;11(1):807.

22. Schulze RJ, Rasineni K, Weller SG, et al. Ethanol exposure inhibits hepatocyte lipophagy by inactivating the small guanosine triphosphatase Rab7. *Hepatol Commun.* 2017;1(2):140-152.

23. Deng X, Pan X, Cheng C, et al. Regulation of SREBP-2 intracellular trafficking improves impaired autophagic flux and alleviates endoplasmic reticulum stress in NAFLD. *Biochim Biophys Acta Mol Cell Biol Lipids.* 2017;1862(3):337-350.

24. Liu Y, Takahashi Y, Desai N, et al. Bif-1 deficiency impairs lipid homeostasis and causes obesity accompanied by insulin resistance. *Sci Rep.* 2016;6:20453.

25. Lettieri Barbato D, Tatulli G, Aquilano K, Ciriolo MR. FoxO1 controls lysosomal acid lipase in adipocytes: implication of lipophagy during nutrient restriction and metformin treatment. *Cell Death Dis.* 2013;4(10):e861.

26. Xu Y, Wang B, Liu X, et al. Sp1 Targeted PARP1 Inhibition Protects Cardiomyocytes From Myocardial Ischemia-Reperfusion Injury via Downregulation of Autophagy. *Front Cell Dev Biol.* 2021;9:621906.

27. Liu JJ, Li Y, Yang MS, Chen R, Cen CQ. SP1-induced ZFAS1 aggravates sepsis-induced cardiac dysfunction via miR-590-3p/NLRP3-mediated autophagy and pyroptosis. *Arch Biochem Biophys.* 2020;695:108611.

28. Huang C, Chen Y, Lai B, Chen YX, Xu CY, Liu YF. Overexpression of SP1 restores autophagy to alleviate acute renal injury induced by ischemia-reperfusion through the miR-205/PTEN/Akt pathway. *J Inflamm (Lond).* 2021;18(1):7.

29. Ling Z, Liu D, Zhang G, et al. miR-361-5p modulates metabolism and autophagy via the Sp1-mediated regulation of PKM2 in prostate cancer. *Oncol Rep.* 2017;38(3):1621-1628.

30. Gaziano JM, Concato J, Brophy M, et al. Million Veteran Program: A mega-biobank to study genetic influences on health and disease. *J Clin Epidemiol.* 2016;70:214-223.

31. Sudlow C, Gallacher J, Allen N, et al. UK biobank: an open access resource for identifying the causes of a wide range of complex diseases of middle and old age. *PLoS Med.* 2015;12(3):e1001779.

32. Nagai A, Hirata M, Kamatani Y, et al. Overview of the BioBank Japan Project: Study design and profile. *J Epidemiol.* 2017;27(3S):S2-S8.

33. Kanoni S, Graham SE, Wang Y, et al. Implicating genes, pleiotropy, and sexual dimorphism at blood lipid loci through multi-ancestry meta-analysis. *Genome Biol.* 2022;23(1):268.

34. Graham SE, Clarke SL, Wu KH, et al. Author Correction: The power of genetic diversity in genome-wide association studies of lipids. *Nature.* 2023;618(7965):E19-E20.

35. Le Bras A. GWAS identifies new blood lipid-associated genetic variants. *Nat Rev Cardiol.* 2018;15(12):728.

36. Teslovich TM, Musunuru K, Smith AV, et al. Biological, clinical and population relevance of 95 loci for blood lipids. *Nature.* 2010;466(7307):707-713.

37. Willer CJ, Schmidt EM, Sengupta S, et al. Discovery and refinement of loci associated with lipid levels. *Nat Genet.* 2013;45(11):1274-1283.

38. Mathieson I. The omnigenic model and polygenic prediction of complex traits. *Am J Hum Genet.* 2021;108(9):1558-1563.

39. Korte A, Farlow A. The advantages and limitations of trait analysis with GWAS: a review. *Plant Methods.* 2013;9:29.

40. Baek Y, Seo BN, Jeong K, Yoo H, Lee S. Lifestyle, genomic types and non-communicable diseases in Korea: a protocol for the Korean Medicine Daejeon Citizen Cohort study (KDCC). *BMJ Open.* 2020;10(4):e034499.

41. Kanai M, Akiyama M, Takahashi A, et al. Genetic analysis of quantitative traits in the Japanese population links cell types to complex human diseases. *Nat Genet.* 2018;50(3):390-400.

42. Ghodsian N, Abner E, Emdin CA, et al. Electronic health record-based genome-wide meta-analysis provides insights on the genetic architecture of non-alcoholic fatty liver disease. *Cell Rep Med.* 2021;2(11):100437.

43. Sollis E, Mosaku A, Abid A, et al. The NHGRI-EBI GWAS Catalog: knowledgebase and deposition resource. *Nucleic Acids Res.* 2023;51(D1):D977-D985.

44. Hemani G, Zheng J, Elsworth B, et al. The MR-Base platform supports systematic causal inference across the human phenome. *Elife.* 2018;7.

45. Sanderson E, Glymour MM, Holmes MV, et al. Mendelian randomization. *Nat Rev Methods Primers.* 2022;2.

46. Purcell S, Neale B, Todd-Brown K, et al. PLINK: a tool set for whole-genome association and population-based linkage analyses. *Am J Hum Genet.* 2007;81(3):559-575.

47. Patel A, Ye T, Xue H, et al. MendelianRandomization v0.9.0: updates to an R package for performing Mendelian randomization analyses using summarized data. *Wellcome Open Res.* 2023;8:449.

48. Wu JH, Lemaitre RN, Manichaikul A, et al. Genome-wide association study identifies novel loci associated with concentrations of four plasma phospholipid fatty acids in the de novo lipogenesis pathway: results from the Cohorts for Heart and Aging Research in Genomic Epidemiology (CHARGE) consortium. *Circ Cardiovasc Genet.* 2013;6(2):171-183.

49. Lee JA, Suh DC, Kang JE, et al. Transcriptional activity of Sp1 is regulated by molecular interactions between the zinc finger DNA binding domain and the inhibitory domain with corepressors, and this interaction is modulated by MEK. *J Biol Chem.* 2005;280(30):28061-28071.

50. Dentin R, Pegorier JP, Benhamed F, et al. Hepatic glucokinase is required for the synergistic action of ChREBP and SREBP-1c on glycolytic and lipogenic gene expression. *J Biol Chem.* 2004;279(19):20314-20326.

51. Park SM, Park M, Ban HJ, et al. Investigation of prodromal features in metabolic syndrome based on transcriptome analysis. *Genes Dis.* 2023;10(3):708-711.

**Supplementary Figures**

**Figure S1.** **eQTL bar plot and GWAS Summary Statistics for SP1 gene-regulating SNPs.** (A) The SNPs regulating the SP1 gene are associated with expression quantitative trait loci (eQTL) relationships in various tissue cells such as adipocytes, brain cells, and blood cells. These SNPs, identified through Genome-Wide Association Studies (GWAS), exhibit a negative effect on Cold Sensitivity. They are in high linkage disequilibrium (LD) with the upstream region of SP1, with an r^2 greater than 0.8. Among these SNPs, only rs7962345 shows a negative effect in terms of eQTL. Therefore, we conducted an analysis focusing on the SNP rs11170510 as a tagging SNP within the same LD block to explore its role in regulating Cold Sensitivity.

**Figure S2. Causal analysis of the effects of cold sensitivity (CS)-associated SNPs on lipid levels.** (A) Directed acyclic graph of the Mendelian Randomization (MR) method. (B) Estimation of the effects of the CS-associated SP1 genotype on blood lipid levels and nonalcoholic fatty liver disease (NAFLD). (C) Scatter plot of the effects of CS-related SNPs on NAFLD. (D) MR effect size for CS-associated SNPs on NAFLD.

**Figure S3. *SP1* upregulation affects mTOR expression.** (A) Analysis of mTOR promoter site. (B) SP1 contributed to phospho-mTOR and mTOR expression. HepG2 cells were transfected with a control vector or *SP1*-expression construct. (E, F) Cell lysates were used for immunoblotting for phospho-mTOR, mTOR, and actin. For densitometry, phospho-mTOR or mTOR levels were normalized to those of actin. Experiments were repeated at least three times. Graphs show mean ± SD. ***p* < 0.01.

**Figure S4. *SP1* upregulation, mediated by gene variants, affects de novo lipogenesis.** (A) Analysis of *SREBP1* promoter site. (B) PBMCs were used for real-time RT-PCR for *SREBP1*, *ACACA,* *SCD*, and *FASN* mRNA (n = 10, reference alleles AA; n = 10, variant alleles GG). Experiments were repeated at least three times. Graphs show mean ± SD. **p* < 0.05, and ***p* < 0.01.

**Figure S5. Analysis of *ACACA, SCD,* and *FASN* promoter sites.** (A) Analysis of *ACACA, SCD,* and *FASN* promoter sites

**Figure S6. SP1 knockdown upregulates autophagy.** (A, C) HepG2 cells were transfected with non-silencing control siRNA (control) or siRNA targeting *SP1* (siSP1). (B, D) Immunofluorescence microscopy analysis of LC3 puncta or LAMP2 staining. Quantification of LC3 puncta or LAMP2 levels in each cell. (E) HepG2 cells were transfected with control siRNA or siSP1, and real-time RT-PCR was performed for *ATG5*, *BECN1*, *ATG16L1*, and *MAP1LC3A* mRNA. Experiments were repeated at least three times. Graphs show mean ± SD. **p* < 0.05, ***p* < 0.01 and *** *p* < 0.001.

**Figure S7. SP1 affects lipid accumulation and lipophagy.** (A) Quantification of BODIPY 493/503 per cell. (B) Quantification of the lipid droplet and LAMP2 merge per cell. Experiments were repeated at least three times. Graphs show mean ± SD. ****p* < 0.001.

**Supplementary Tables captions**

**Table S1. Demographic characteristics and lipid measurements in the KDCC cohort samples.** We analyzed the average distribution of basic characteristics and measured lipid metabolism-related parameters, such as total cholesterol, low-density lipoprotein-C (LDL-C), high-density lipoprotein-C (HDL-C), and triglycerides, in a cohort of 2,000 individuals from the KDCC. Additionally, we examined the mean and standard deviation of each phenotype for the group with *SP1* variant alleles (n = 118) and the group with the *SP1* reference alleles. Furthermore, we investigated the correlation (r) between cold sensitivity (CS) and each phenotype. Within the cohort, we calculated the mean and standard error values for the top and bottom 10% of CS separately for males and females, followed by *t*-tests to explore potential differences between these groups.

**Table S2. Genes in the lipogenesis pathway showing differential expression according to the presence of the reference alleles and variant alleles of *SP1*.** In the KDCC cohort, we performed RNA-seq on 100 PBMC samples from a total of 2,000 individuals to investigate gene expression. We compared the average read counts of genes associated with the lipogenic pathway between the group bearing *SP1* variant allele types and the group bearing the reference allele types. After excluding low signals with read counts less than 5, we found significant differences in the expression levels of 14 genes (*p* < 0.05).

**Table S3.** **Genes related to autophagy showing differential expression according to the presence of the reference alleles and variant alleles of *SP1*.** We analyzed the average read counts from RNA-seq experiments for genes related to the autophagy pathway, taking into account the allele types of *SP1*. After removing low signals with read counts less than 5, we found significant differences in the expression levels of 16 genes (*p* < 0.05).
